# Supplementary material for: Mast Cells Retard Tumor Growth in Ovarian Cancer: Insights from a Mouse Model
Source: Cancers (Basel). 2023 Aug 26;15(17):4278. doi: 10.3390/cancers15174278 (PMC10487127; doi:10.3390/cancers15174278)
Supplement: Supplementary file 1 [file cancers-15-04278-s001.zip › cancers-2569220-supplementary.pdf]

Supplemental Material

Mast Cells Retard Tumor Growth in Ovarian Cancer: Insights from a Mouse Model

Nicole Meyer [1,2,3], Nicole Hinz [1], Anne Schumacher [1,2,3], Christine Weißenborn [1], Beate Fink [2], Mario Bauer [2], Sophie von Lenthe [1], Atanas Ignatov [1], Stefan Fest [1,2,4] and Ana Claudia Zenclussen [1,2,3,*]

[1] Experimental Obstetrics and Gynecology, Medical Faculty, Otto-von-Guericke University Magdeburg, 39108 Magdeburg, Germany;
[2] Department of Environmental Immunology, UFZ-Helmholtz Centre for Environmental Research Leipzig-Halle, 04318 Leipzig, Germany
[3] Perinatal Immunology, Saxonian Incubator for Clinical Translation (SIKT), Medical Faculty, Leipzig University, 04103 Leipzig, Germany
[4] Department of Pediatrics, Städtisches Klinikum Dessau, Academic Hospital of University Brandenburg, 06847 Dessau-Rosslau, Germany
* Correspondence: ana.zenclussen@ufz.de; Tel.: +49-341-6025-1265

Suppl. Table S1. *RT-qPCR primers.*

Gene	Forward Primer	Reverse Primer	UPL Number
Actb	aaggccaaccgtgaaaagat	gtggtacgaccagaggcatac	56
Adam10	gggaagaaatgcaagctgaa	ctgtacagcagggtccttgac	38
Ccl2	gtccctgtcatgcttctgg	cgttaactgcatctggctga	19
Ccl3	ttgaaaccagcagcctttg	gccggtttctcttagtcagga	20
Ccl4	cctctctctcctcttgctcgt	ggagggtcagagcccatt	1
Ccl5	tgcagaggactctgagacagc	gagtggtgtccgagccata	110
Ccl11	agagctccacagcgcttct	gcaggaagttgggatgga	18
Ccl17	tgcttctggggacttttctg	gaatggcccctttgaagtaa	27
Ccl22	caagcctggcgttgtttt	cagacccatggtcatcagg	52
Cpa3	gctattaattccttatggctacacatt	gtggcaatccttgcaacttt	16
Csf2	gcatgtagaggccatcaaaga	cgggtctgcacacatgtta	79
Cxcl1	gactccagccacactccaac	tgacagcgcagctcattg	83
Cxcl5	ttcttgggtgtgttaagagtgttc	tctgcatgacacagcagctt	26
Cxcl12	ccaaactgtgcccttcagat	atttcgggtcaatgcacact	41
Cxcl13	cagaatgaggctcagcacag	atgggcttccagaataccg	63
Cxcr4	gcactcaactccatgagcag	cgtggagacggaagagtgtc	19
Cxcr6	agctactgggcttctcttctga	gacctccttgaactttaggaagc	2
Fasl	accggtggtatttttcatgg	tttaaggctttggttggtgaa	21
Fcer1a	tgtgtacttgaatgtaacgcaaga	tggactaagaccatgtcagca	68
Flt1	ggcccgggatatttataagaac	ccatccattttaggggaagtc	55
Gapd	gggttcctataaatacggactgc	ccattttgtctacgggacga	52
Gypc	cccaccacagtgctaccc	ggacttccctgggttaggc	70
Ifna1	ggatgtgaccttcctcagactc	tccaaagtccttcctgtcctt	51
Ifng	cattcagagctgcagtgacc	ctgtctggcctgctgttaaa	52
Il3	cgtctctcctaaccgtggaa	tgggctacttcgattttggt	13
Il4	ggtctcaacccccagctagt	tggatatggctcctggtacat	15

Il5	acattgaccgccaaaaagag	atccaggaactgcctcgtc	91
Il10	cagccgggaagacaataact	gttgtccagctggtcctttg	48
Il11	tactccgccgtttacagctc	caggggggatcacaggttg	27
Il12a	ccaggtgtcttagccagtcc	gcagtgcaggaataatgtttca	62
Il13	acccagaggatattgcatgg	tgggctacttcgattttggt	19
Il23	gagcaacttcacacctcccta	tagaactcaggctgggcatc	19
Lgals1	ccaaggaagatgggacctg	aggtcagcctggtcaaagg	11
Lgals9	ccaggggactaccaagagttt	cttcgtgttgcaaaccacat	12
Mcpt1	gcaggccctactattcctga	ccgtgatgatcttcagatgg	50
Mcpt2	gcacttcttttgccttctgg	cgaaccgttcttagtggtgaa	73
Mcpt4	tccttatggcacttctcttgc	gccatgtaagggcgagaat	45
Mcpt5	aggatgcatcttcttactcttcatc	cgtgcctccaatgatctctc	68
Mcpt6	tgctgtgtgctggaaatacc	cccttcactttgcagacca	51
Mcpt7	gtgtgctgggaatgaaggac	ttctaccttgcagaccagagg	51
Mcpt8	ggatgttcctgctcctggt	tggggtttggactctgtacc	83
Mcpt9	atggcacttctcttgccttc	gtcgggagtgtggttcagac	19
Mmp2	aactttgagaaggatggcaagt	tgccacccatggtaaacaa	29
Mmp9	acgacatagacggcatcca	gctgtggttcagttgtggtg	19
Mmp12	ccacttcgccaaaaggttta	ggggtaagcagggtccat	51
Par1	gtcttcccgcgtccctat	gggttcaccgtagcatctgt	20
Par2	ggggatgcgaagtctcag	agacttcttcctttactgttgttgc	3
Rplp0	ctgctgaacatgctgaacatc	tgtcgagcacttcagggtta	62
Scf	agcgctgcctttccttatg	cgcagatctccttggttttg	68
Serpine1	aggatcgaggtaaacgagagc	gcgggctgagatgacaaa	69
Stat3	cgatgcctgtgggaagag	gtcactacggcggctgtt	25
Timp1	gcaaagagctttctcaaagacc	agggatagataaacagggaaacact	76
Timp2	ttttgcaatgcagacgtagtg	ggaatccacctccttctcg	21
Timp3	cacggaagcctctgaaagtc	tcccacctctccacaaagtt	62
Tnf	tcttctcattcctgcttgtgg	ggtctgggccatagaactga	49
Trp53	acgcttctccgaagactgg	agggagctcgaggctgata	25
Ubc	gtctgctgtgtgaggactgc	cctccagggtgatggtctta	77

Fig. S1

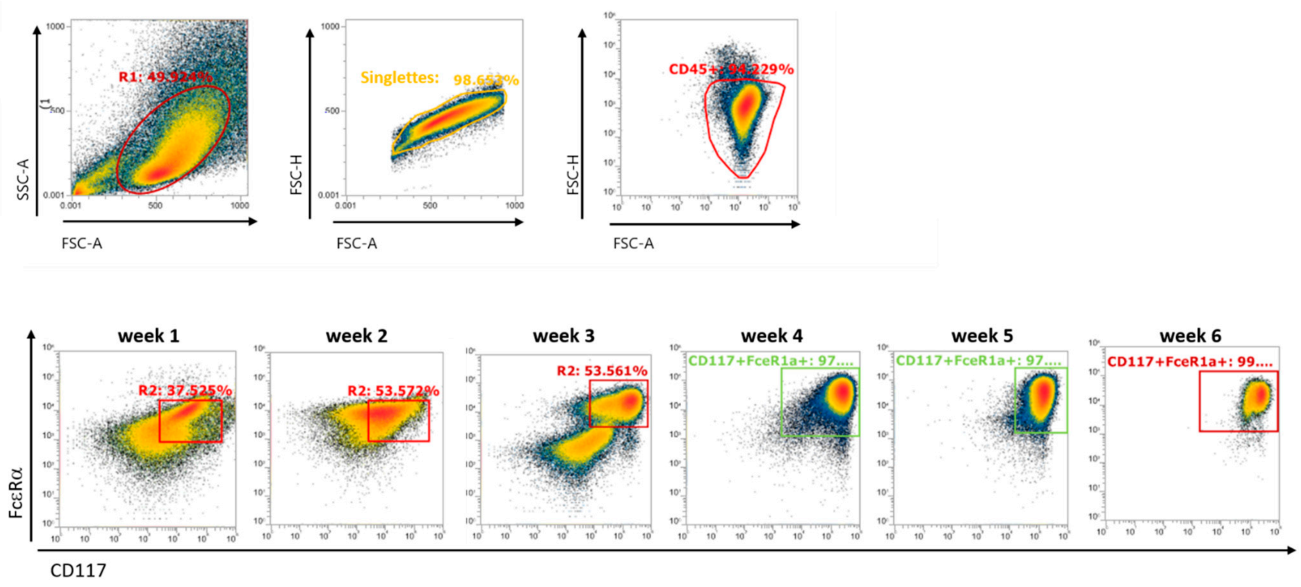

Figure S1: Purity of cultured BMMCs. Representative dot plots from cultured BMMCs used for systemic transfer into Kit$^{W\text{-}sh}$ mice. Cells were stained with antibodies against FcεRα and CD117. BMMCs showed a purity of >99% after 6 weeks in culture. BMMC. bone morrow-derived mast cells.

Suppl. Table S2. *RT-qPCR results.*

**Gene**	**ID8 in C57BL/6J** (mean ± SD)	**ID8 in Kit$^{W\text{-}sh}$** (mean ± SD)	**BMMC + ID8 in Kit$^{W\text{-}sh}$** (mean ± SD)
Csf2	4.14 ± 3.16	4.33 ± 2.45	6.67 ± 3.81
Ifnβ1	7.91 ± 3.69	4.33 ± 1.29	9.43 ± 4.36
Ifnγ	7.93 ± 3.76	4.13 ± 1.61	10.63 ± 3.45
Il3	7.36 ± 3.49	2.98 ± 0.85 *	9.67 ± 3.98
Il4	7.63 ± 3.22	4.45 ± 1.28	10.13 ± 3.45
Il5	2.39 ± 2.84	3.75 ± 1.09	5.90 ± 3.40
Il10	5.98 ± 2.56	7.48 ± 1.24	8.47 ± 4.90
Il11	4.68 ± 3.04	6.28 ± 2.42	6.20 ± 5.42
Il12a	6.20 ± 2.71	6.65 ± 2.40	8.90 ± 3.69
Il13	3.68 ± 2.83	4.90 ± 1.32	5.30 ± 5.33
Il23	1.44 ± 1.81	5.68 ± 1.23 **	5.20 ± 5.25
Tnf	7.54 ± 2.56	8.35 ± 1.44	10.50 ± 4.61
Ccl2	10.84 ± 2.96	11.60 ± 1.95	13.73 ± 3.94
Ccl3	8.40 ± 3.06	8.43 ± 1.73	11.60 ± 3.86

Ccl4	8.70 ± 2.74	9.38 ± 1.72	12.07 ± 4.27
Ccl5	10.93 ± 2.62	10.43 ± 1.51	15.83 ± 3.16
Ccl11	8.58 ± 2.69	8.40 ± 1.56	11.47 ± 3.92
Ccl17	10.66 ± 3.82	10.58 ± 0.82	14.77 ± 3.30
Ccl22	8.01 ± 2.63	8.50 ± 1.52	12.13 ± 3.99
Cxcl1	7.70 ± 2.85	10.88 ± 2.27	10.17 ± 6.64
Cxcl5	10.13 ± 3.30	12.50 ± 2.46	11.67 ± 4.79
Cxcl13	10.96 ± 2.99	10.85 ± 0.93	15.67 ± 3.35
Cpa3	11.99 ± 3.54	3.45 ± 1.36 ***	6.47 ± 7.01
FceRa	8.07 ± 3.17	3.88 ± 1.63	5.67 ± 5.12
Mcpt1	6.61 ± 4.27	2.13 ± 1.52	0.33 ± 0.47 **
Mcpt2	8.83 ± 3.60	2.73 ± 0.87 **	3.00 ± 4.24
Mcpt4	10.49 ± 2.42	1.68 ± 0.90 ****	6.17 ± 6.59
Mcpt5	11.11 ± 2.16	3.70 ± 0.44 ****	5.60 ± 7.92
Mcpt6	13.17 ± 2.18	4.50 ± 0.38 ****	10.40 ± 6.05
Mcpt7	10.07 ± 2.92	0.50 ± 0.50 ****	8.73 ± 4.69
Mcpt8	1.88 ± 2.75	5.65 ± 3.75	0.00 ± 0.00
Mcpt9	0.91 ± 1.20	0.35 ± 0.61	2.80 ±3 .96
Scf	10.36 ±2.36	11.65 ± 1.31	12.80 ± 4.24
Adam10	9.71 ± 2.31	11.40 ± 1.30	11.23 ± 4.56
Cxcl12	12.77 ± 2.88	13.83 ± 1.32	15.03 ± 4.03
Cxcr4	9.74 ± 2.98	10.35 ±1.32	11.97 ± 3.87
Cxcr6	5.34 ± 2.80	7.10 ± 1.51	7.70 ± 4.69
Fasl	4.13 ± 2.56	4.73 ± 1.88	7.43 ± 4.40
Flt1	9.18 ± 2.98	10.00 ± 0.85	11.63 ± 4.24
Gypc	7.36 ± 2.06	9.33 ± 1.37	10.67 ± 4.09
Ifn1	9.13 ± 3.79	3.95 ± 1.98 *	10.33 ± 4.17
Lgals1	17.89 ± 3.43	18.40 ± 1.51	20.87 ± 3.79
Lgals9	12.51 ± 2.43	14.48 ± 1.44	13.83 ± 6.43
Lgals3	10.14 ± 2.18	11.60 ± 1.16	12.93 ± 4.48
Mmp2	12.93 ± 2.20	15.45 ± 1.56	15.13 ± .52
Mmp9	8.54 ± 4.07	10.68 ± 2.05	10.33 ± 4.17
Mmp12	10.19 ± 4.91	9.88 ± 1.61	12.83 ± 3.44
Serpine1	10.33 ± 3.12	11.63 ± 1.95	13.10 ±4.39
Par1	12.28 ± 3.24	13.50 ± 1.42	14.67 ± 4.54
Par2	8.92 ± 2.51	9.18 ± 1.12	9.80 ± 5.83
Stat3	11.49 ± 2.59	12.85 ± 1.44	13.57± 4.54
Timp1	13.60 ± 3.80	15.23 ± 2.0	16.17 ± 4.06
Timp2	15.50 ± 2.78	16.45 ± 1.19	17.47 ± 4.29
Timp3	15.22 ± 2.31	17.70 ± 1.57	17.43 ± 5.14
Trp53	10.54 ± 2.33	12.48 ± 1.29	12.70 ± 5.00

* compared to C57BL/6J (*p<0.05, **p<0.01, ***p<0.001)
